# Association of Prenatal Maternal Anemia with Tics and Tourette’s Syndrome in Offspring

**DOI:** 10.3390/jpm11101038

**Published:** 2021-10-17

**Authors:** Yi-Chun Liu, Vincent Chin-Hung Chen, Yin-To Liao, Yi-Lung Chen

**Affiliations:** 1 Department of Psychiatry, Changhua Christian Children’s Hospital, Changhua 500, Taiwan; 183711@cch.org.tw; 2 Department of Psychiatry, Changhua Christian Hospital, Changhua 500, Taiwan; 3 Department of Healthcare Administration, Asia University, Taichung 413, Taiwan; 4 School of Medicine, Chang Gung University, Taoyuan 333, Taiwan; cch1966@gmail.com; 5 Department of Psychiatry, Chiayi Chang Gung Memorial Hospital, Chiayi 613, Taiwan; 6 Department of Psychiatry, Chung Shan Medical University and Chung Shan Medical University Hospital, Taichung 402, Taiwan; je2tezy@yahoo.com.tw; 7 Department of Psychology, Asia University, Taichung 413, Taiwan

**Keywords:** tics, Tourette’s syndrome, prenatal anemia, maternal anemia, iron deficiency anemia

## Abstract

Iron deficiency anemia (IDA) accounts for most of the anemia in pregnancy, and iron is essential for neurodevelopment. Tics and Tourette’s syndrome (TS) are neurodevelopmental disorders that manifest in childhood. A few studies reported an inconclusive association between iron deficiency and tics in children. No study has investigated the relationship between prenatal maternal anemia and tics in children. We aimed to assess the relationship between prenatal anemia exposure and the incidence of tics or TS in offspring. We linked the Taiwan National Health Insurance Research Database to the Maternal and Child Health Database for the analysis and identified 153,854 children with prenatal anemia exposure and 2,014,619 children without prenatal anemia exposure from 2004 to 2016 and followed them through 2017. Cox regression models were applied to compare the risk of tics or TS between the exposed and nonexposed groups. Among the exposed group, 37,832 were exposed at ≤12 weeks of gestational age (GA) and 116,022 at >12 weeks of GA. We observed an increased risk of tics and TS in those exposed at ≤12 weeks compared with the nonexposed group (adjusted hazard ratio (aHR) = 1.23, 95% confidence interval (CI): 1.12–1.34). The result remained consistent after adjusting for birth year, sex, birth order, maternal age, low-income levels, gestational age, birth weight, and alcohol use and smoking during pregnancy (aHR = 1.16, CI: 1.04–1.28). Fetuses exposed to maternal anemia at ≤12 weeks of GA are at high risk of tics or TS. However, this effect was attenuated to insignificance in the sibling comparison. Our study highlights the importance of detection of anemia during pregnancy and proper timing of iron supplementation.

## 1. Introduction

Anemia is common during pregnancy and is a globally recognized health problem. Because of hemodilution and increased iron requirements for fetal and placental growth, iron deficiency often occurs and is the leading cause of anemia in pregnancy [1,2]. Iron deficiency anemia (IDA) is estimated to account for up to 75% of all anemia during pregnancy [3]; hence, the World Health Organization recommends routine daily oral iron supplementation during pregnancy to prevent IDA. The source of fetal iron depends mainly on maternal iron transport through the placenta [4,5]. Iron is essential for fetal growth, and maternal IDA can cause premature birth and low birth weight [4]. Iron is also critical for aspects of brain development such as myelination [6], glial integrity [7], neurotransmitters [6], neurogenesis [8], and energy metabolism [9]. In animal models, early iron deficiency in the developing brain caused striatal dysfunction and persistent behavioral changes, including involuntary movements and tic-like behaviors [7,10,11].

Tics and Tourette’s syndrome (TS) are neurodevelopmental disorders that often manifest in childhood and peak in adolescence [12]. Tics are involuntary, sudden, rapid, and repetitive vocalizations or movements [12,13]. TS is defined by the simultaneous presence of multiple motor and at least one or more vocal tics for over 1 year [14]. The exact etiology and pathogenesis of tics and TS have not yet been identified. The heritability of tics and TS was estimated to be 0.25–0.77 [15,16]. Tics and TS are commonly considered to be polygenic diseases that are influenced by multiple environmental factors [17,18]. The involvement of cortico-basal ganglia circuits, particularly alterations in the striatal dopaminergic pathway, has been postulated to be central to the pathophysiology of tic disorder [12,13,19]. In one rat model study in 2014, researchers injected a specific local GABA-A receptor antagonist into the dorsal striatum of adult rats that induced tic movements [13]. Similarly, another in vivo study in juvenile rats in 2018 revealed that early destruction of the dorsal striatum and acquired alterations in dopaminergic levels eventually led to tic behaviors [12]. A longitudinal prospective study focusing on images of the human brain also identified an association between a reduction in striatum volume and the presence of tic symptoms [20]. Several trace elements and nutrients have been shown to play an important role in the production and regulation of dopamine, which may further interact with the striatal dopaminergic pathway [21,22,23]. One observation study by Qian et al. reported a reversed relationship between human zinc, copper, and iron blood levels and the incidence of TS [24]. In addition, two studies observed a correlation between vitamin D levels and the prevalence and severity of tics in children [25,26].

The developing brain is particularly sensitive and susceptible to prenatal environmental events, as has been widely proven through experimental animal models [27]. Moreover, despite the limited experimental feasibility in humans, various observational studies have linked prenatal environmental injury to subsequent long-term brain development, even after birth [28,29]. The preliminary structures of the brain and the elementary compartments of the central nervous system are typically determined by the end of the eighth week of gestation [29]. In Taiwan, hemoglobin levels are screened at least once throughout pregnancy (at 12 weeks of gestation) and additional measurements are taken if abnormalities exist that require follow-up. Therefore, to explore the effect of anemia on very early brain development, we distinguish between anemia diagnosed before and anemia diagnosed after the 12 weeks of gestation for comparison in this study.

To the best of our knowledge, no study has examined the association between prenatal exposure to anemic conditions and the subsequent occurrence of tics. This study, therefore, employed a retrospective cohort approach based on a national insurance database to investigate the association between prenatal anemia exposure and the incidence of tics in offspring. To minimize the interference of preterm births, which may have neurodevelopmental abnormalities, we included only births at term in our follow-up cohort. We hypothesized that prenatal anemia exposure would increase the risk of tics in offspring and that the earlier the exposure, the more pronounced the effect would be.

## 2. Materials and Methods

### 2.1. Study Population

**Figure 1:**
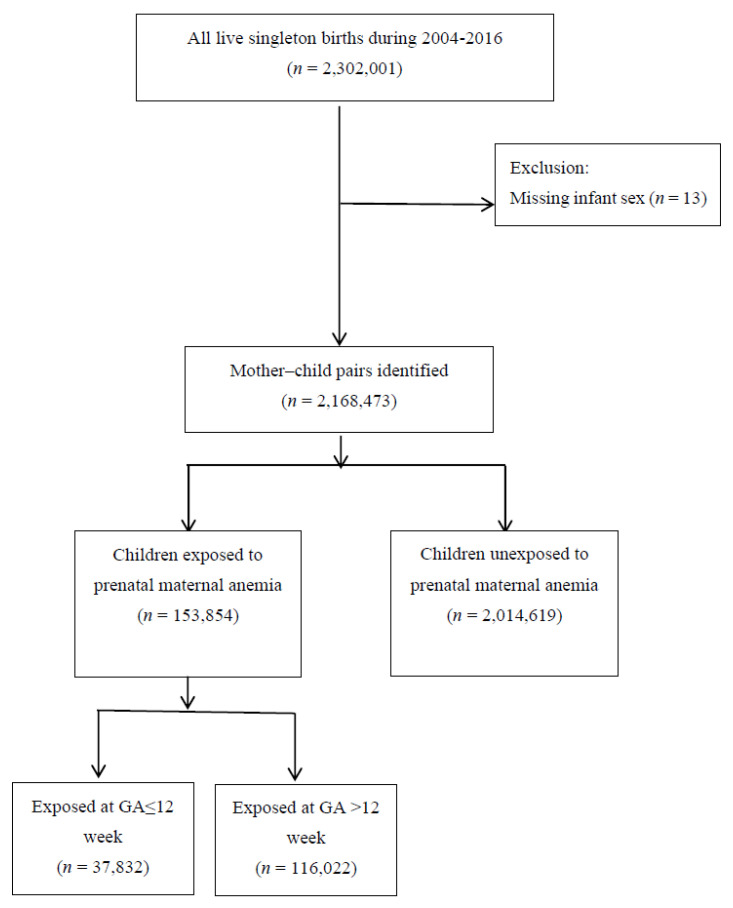
Overview of the design of this study (GA: gestational age).

The Taiwanese government launched a single-payer nationwide insurance program, the National Health Insurance (NHI), on March 1, 1995. The NHI has covered 99.6% of the national population as of December 2010 [30]. The Bureau of the NHI extracted medical claims data and de-identified personal information to form the National Health Insurance Research Database (NHIRD). Our study sample was derived mainly from the NHIRD. This database provides records of inpatient and outpatient hospital care, ambulatory services, medication prescriptions, prescription dates, and diagnosis codes. We also used data from the Taiwan Birth Certificate Registry between 1 January 2004 and 31 December 2016 to ascertain all live singleton births and obtain their birth dates and gestational age information. We included only singleton births because multiple births inherently predispose fetuses to anemia and intrauterine growth restriction, which may mediate the relationship between anemia and brain development [31,32]. The linkages between these two datasets were thus used to determine prenatal anemia exposure during the first trimester or before pregnancy. Further data from the Taiwan Maternal and Child Health Database [33] enabled us to obtain complete information on children, their fathers, and maternal anemia exposure over the 2004–2016 period. Figure 1 presented the flow chart of our study design. This study was approved by the Research Ethics Committee of the China Medical University and Hospital (approval number: CMUH108-REC1–142).

### 2.2. Exposure Measure

We identified cases of maternal anemia according to the International Classification of Diseases, Ninth Revision, Clinical Modification (ICD-9-CM) codes 648.2, 280, 285.9, and 285.8 and ICD-10-CM codes O99.0, O99.81, D50, D64.9, and D64.89. The above diagnostic codes include IDA, anemia complicating pregnancy, and anemia without specific causes. Mothers with congenital disorders known to be associated with hereditary hemolytic anemias, including thalassemia and sickle-cell disease (ICD-9-CM code 282; ICD-10-CM codes D55, D56, D57, and D58), were excluded. At least one inpatient or outpatient diagnosis of maternal anemia from 1 January 2004 to 31 December 2016 was required for inclusion in the study. To compare the critical windows of exposure to prenatal anemia, we further divided the exposure time into two periods: (1) ≤12 weeks of gestational age (GA); and (2) >12 weeks of GA (Figure 1). The date of conception was calculated using the date of delivery and the GA from the maternity records.

### 2.3. Outcome Measures

All children with and without prenatal anemia exposure were followed from birth to 31 December 2017 or until death. The primary outcomes were incident diagnoses of tics or TS. The diagnoses were ascertained on the basis of at least one inpatient record or at least three outpatient diagnoses based on ICD-9-CM code 307.2 and ICD-10-CM code F95.

### 2.4. Covariates

The covariates included sex, GA, birth weight, age of the child (by the end of the study period), birth order, maternal age at index birth, alcohol use and smoking during pregnancy, and low-income level. Low-income level was determined using the Low-Income and Middle–Low-Income Households dataset from the government welfare databases [34].

### 2.5. Statistical Analysis

We first examined the demographic characteristics of the whole sample. Then, we performed an analysis of the characteristics of the two groups by different periods of maternal anemia (i.e., ≤12 weeks of GA and >12 weeks of GA). To determine the effect of maternal anemia on tic disorders, we made two comparisons: a whole-population comparison and a sibling comparison. The whole-population comparison group comprised children born to mothers with and without anemia. Cox regression and proportional hazard analyses were used to estimate the risks of tic disorders. In model 1, we adjusted only for birth year and sex. In model 2, to control for possible confounders, we adjusted for birth year, sex, low-income level, maternal age at birth, birth order, birth weight, and alcohol use and smoking during pregnancy. We repeated the analysis after categorizing the exposure by time of diagnosis of maternal anemia (i.e., ≤12 weeks of GA and >12 weeks of GA). We used the sibling comparison to automatically match some unmeasured confounding factors such as gene liability and background environment. For the sibling comparison, within-family analysis was conducted using conditional logistic and Cox regression models. We used the biological mother as the stratum variable to compare risks of tic disorders between exposed and unexposed siblings. We also adjusted for birth year, sex, maternal age at childbirth, birth order, GA, birth weight, and alcohol use and smoking during pregnancy. In these analyses, invariant variables related to the biological mother (e.g., low-income level) remained constant in each stratum and were therefore not included as covariates. These analyses were stratified by different exposure periods to maternal anemia. Adjusted hazard ratios and their 95% confidence intervals (CI) and *p*-value were calculated. SAS 9.4 was used for all statistical analyses.

## 3. Results

### 3.1. Characteristics of Offspring with or without Prenatal Anemia

We identified all single live births (*n* = 2,302,001) from the Taiwanese Birth Certificate Registry. We excluded 13 cases because of missing data on the infant’s gender. Since the Taiwanese Birth Certificate Registry database only contains the national identification number of the woman who gave birth, we further linked the Taiwanese Birth Certificate Registry database to the Taiwan Maternal and Child Health Database and used the mother’s national identification number, the child’s gender, and their year of birth as key variables to obtain the national identification number of the child. Through this process, we determined the mother–child pairing (*n* = 2,168,473). The pairing comprised 153,854 children exposed to prenatal maternal anemia and 2,014,619 unexposed children. Among the exposed group, 37,832 children were exposed in early GA, defined as ≤12 weeks, and 116,022 children in later GA (>12 weeks). Table 1 lists the mean and standard deviation and proportion of characteristics of children exposed to prenatal maternal anemia and children unexposed (for details of the selected characteristics between groups, please see Supplementary Table S1). In both groups, the gender distribution was slightly skewed, with a predominance of males. In the exposed and nonexposed groups, 51.49% and 52.00%, respectively, were male. The mean age was 5.49 ± 3.63 years in the exposed group and 6.75 ± 3.71 years in the nonexposed group.

### 3.2. Association between Maternal Anemia and Tics or TS in Offspring

We observed no statistically significant differences when comparing solely the risk of tics between the exposed and nonexposed groups, either in the crude model or in the adjusted models (Table 2). After stratifying exposure by time of diagnosis of anemia, we observed that offspring of mothers with an early (≤12 weeks of GA) diagnosis of anemia had an increased risk of tics or TS (adjusted hazard ratio (aHR), 1.23; 95% CI, 1.12–1.34, *p* < 0.001) in the whole-population comparison model, which was adjusted only for birth year and sex (Table 2). In the advanced adjusted model, which included additional socioeconomic and birth factors, the association between maternal anemia (≤12 weeks of GA) and the risk of tics or TS was attenuated but still reached statistical significance (aHR, 1.16; 95% CI, 1.04–1.28, *p* = 0.008) (Table 2). In the sibling comparison analysis, 64,741 discordant pairs were identified out of the 145,096 participants in the analysis. The results reveal no significant increase in the risk of tics and TS in the offspring of mothers diagnosed with anemia at ≤12 weeks of GA (aHR, 1.06; 95% CI, 0.80–1.32, *p* = 0.685).

## 4. Discussion

In this nationwide population-based study, we observed an association between maternal anemia at ≤12 weeks of GA and the risk of tics or TS in offspring. The association remained significant after adjusting for income status, maternal age, sex, GA, birth order, and alcohol use and smoking during pregnancy. In the analysis of discordant sibling pairs with a relatively limited number of participants, the effect of early prenatal anemia exposure on the risk of tics and TS diminished to insignificance. To the best of our knowledge, this is the first study to explore the relationship between prenatal maternal anemia and tics or TS in offspring.

Early life iron deficiency is associated with a number of cognitive, motor, and emotional disorders, as well as other neurodevelopmental disorders [8]. Only a few studies have reported an association between iron deficiency and tics, and our results are consistent with previous research results. A study of individuals with TS of any age reported that ferritin and serum iron levels were lower in the TS group than in the control group [35]. Ferritin level was associated with smaller basal ganglia volume in the TS group, but not with severity of symptoms. This study included a smaller sample size of 63 children and adults with TS aged 7–57 years. Sampling and recruitment from a child study center and a local TS association may have led to missing participants with severe iron deficiency. Iron deficiency during critical periods of neurodevelopment in children, such as gestation or infancy, was not reflected in the present blood chemistry laboratory tests. These might explain why reduced ferritin levels did not correlate with the severity of TS symptoms. Our study used the mother–child linkage to explore the effect of iron deficiency (ID) in early life and the later risk of TS. We used anemia as a surrogate marker of ID, as in a previous study [36], and defined a subgroup GA ≤ 12 weeks to indicate ID in the embryonic period. Our results indicate that mothers with gestational anemia at ≤12 weeks of GA had a slightly increased risk of tics in their offspring, with an aHR of 1.16, after adjusting for additional socioeconomic and birth factors. However, we did not observe such an association in the case of gestational anemia at GA > 12 weeks. These attenuated associations of ID and TS may be due to the inclusion of gestational anemia unrelated to ID or the lack of adjustment for confounders of tic disorders (for example, maternal smoking, small size for gestational age, and obstetric delivery) [37]. A neuroimaging study compared serum ferritin levels and T2 MRI values in 14 adult patients with TS and 14 healthy controls. The results indicated that lower ferritin in the TS group was associated with an altered T2 relaxation time in the putamen and insular cortex [38]. The study included a small sample size of adults with TS, which may have resulted in low statistical power. Furthermore, symptoms of TS tend to remit with age, which makes it difficult to generalize the results to children. A retrospective study revealed that iron supplementation improved the severity of tic symptoms in iron-deficient and iron-sufficient groups (n = 5 and 10, respectively) [39]. However, because of the study’s naturalistic design and small sample size, the results should be interpreted with caution. Moreover, the design of the above studies was cross-sectional or retrospective; hence, the causality between ID and TS or tics was inconclusive. In our study, we used a population-based database and cohort design to avoid selection bias, reverse causality, and recall bias as much as possible. The use of a large and representative nationwide population reduced type II errors. A population-based study by Chen et al. indicated an increased risk of tic disorder in children and adolescents with IDA (OR = 1.70, CI 1.03–2.78) [40]. Girls and adolescents with IDA were more prone to tics with ORs of 2.95 and 3.73, respectively [40]. The study extracted data comprising 1,000,000 individuals from the NHIRD and included children and adolescents with diagnoses of IDA. Despite the large sample size, it is difficult to confirm causality because the study focused on individuals with ID after birth and did not adjust for various known risk factors for tics (e.g., preterm labor, birth weight, multiple births, and parity) [37,41]. Our study focused specifically on ID during neurodevelopmental periods (e.g., during pregnancy) and the effect on the risk of tics or TS in offspring, aiming to determine causality. We used data from a whole-population registry, along with mother–child linkages from the NHIRD, and we also analyzed discordant sibling pairs to discern common factors shared by siblings. Our results reveal a diminished risk of association of tics or TS with prenatal maternal anemia when genetic inheritance and shared environmental factors were adjusted for. Environmental factors, such as iron status, can interact with gene expression to some extent [23]. However, as shown in the previous report, genetic inheritance itself has a strong impact on the pathogenesis of tics and TS [16]. On the other hand, whether this result implies that the combined effects of shared genetic and environmental background outweigh the effect of maternal anemia alone remains uncertain. This is because the sample population of the sibling analysis was smaller than that in the whole-population comparison model. In addition, we could neither confirm whether anemic mothers had ever received iron supplements nor confirm the effect of the iron supplements during pregnancy. A common scenario in clinical practice is that a pregnant woman is more aware of anemia screening and iron supplements during her subsequent pregnancies because she had been diagnosed with anemia in her previous pregnancy. A number of potential unshared confounders (health-seeking behaviors, nutrient supplements, trace element status, and diet habits) between one birth and another of the same mother may lead to inconsistent results between sibling discordant comparisons and the original unpaired comparisons [42]. Even in the absence of confounders, the results of discordant sibling studies could still have been attenuated by random measurement errors or so-called ‘misclassification’ in our study [42].

As reported in previous studies, mothers with low ferritin levels were more likely to give birth to infants with low ferritin levels [43,44]. This would indicate that the fetus’s iron stores were compromised by maternal ID. It is critical for the fetus to obtain adequate iron stores from the mother because of the relatively low and inadequate absorption of iron in the infant’s digestive system [45]. Low maternal serum ferritin not only hinders the fetus from accumulating iron but also jeopardizes the later development of iron homeostasis in the infant [8]. The timing of ID during gestation is also crucial. An animal study with rats revealed that maternal ID before pregnancy and in the first third of the gestation period had a negative effect on iron stores in the brain, whereas ID in the last third of pregnancy did not impair fetal neurodevelopment [46]. Therefore, ID in different stages of the perinatal period may affect brain development differently. In humans, the association between perinatal ID, neurodevelopment, and risk of tics or TS remains unexplored. Further comprehensive neuroimaging studies with consideration of the timing of ID and adjustment for risk factors for tics (e.g., smoking, labor presentation, GA) will help us to elucidate the influence of genetic and environmental factors on tics and TS.

This study has some limitations, and the negative findings should be interpreted with caution before presuming the effect of maternal anemia or ID on tics or TS. Several methodological factors need to be considered. First, the validity of tics or TS diagnoses was limited, and the insurance claims data did not include symptom severity. Although board-certified psychiatrists or neurologists made the diagnoses, which improved the diagnostic reliability, no studies were conducted to verify the validity of the diagnoses. Second, some covariates, such as labor presentation, the body mass index of mothers, and environmental information, are not documented in the NHIRD; hence, their effect on tics or TS risk could not be evaluated. In addition, the prevalence of smoking during pregnancy in the NHIRD was about 7 per 10,000, which was significantly lower than the prevalence of smoking among pregnant women in the previous study on the Taiwanese birth cohort [47]. In the previous report, the prevalence of smoking among pregnant mothers ranged from 2.8 to 3.5%. It is reasonable to infer that pregnant women tend to under-report the rate of substance use, leading to an underestimation of alcohol and smoking prevalence in our database. The apparent underestimation may lead to a poor adjustment for these two covariates in our study [47]. Third, we categorized the timing of maternal ID as ≤12 weeks of GA and >12 weeks of GA according to the national screening guidelines for hemoglobin levels in pregnant women rather than using more precise periods of embryonic development and conducting laboratory tests for maternal ferritin levels. Fourth, we extended the follow-up period to 13 years to better include the most common age when tics and TS are prevalent. However, given the nature of the insurance claims data of the NHIRD, those who had tics or TS but did not seek any medical help were not included in this study. A prospective clinical study incorporating higher diagnostic accuracy and laboratory tests and designed to confirm the effect of maternal ID on tics and TS risk in offspring should overcome these problems. However, in practice, a large sample size with a long follow-up period might be difficult to achieve.

## 5. Conclusions

In this study, maternal anemia at ≤12 weeks of GA was found to increase the risk of tics and TS in offspring. This effect was attenuated when shared genetic and environmental factors were considered. Our findings provide insight into both the effects of ID according to gestational age and the role of the proper timing of iron supplementation in pregnancy. Monitoring for possible tic or TS symptoms in offspring previously exposed to maternal ID might facilitate early intervention. Further cohort studies with comprehensive laboratory and neuroimaging examinations are required to clarify the causal relationship between early life ID and the risk of tics and TS.

## Figures and Tables

**Table 1:** Selected characteristics by maternal anemia exposure in the study cohort.

	All Exposed			Unexposed
	(*n* = 153,854)	Exposed, GA ^1^ ≤12 weeks (*n* = 37,832)	Exposed, GA ^1^ >12 weeks (*n* = 116,022)	(*n* = 2,014,619)
	Mean (SD)	Mean (SD)	Mean (SD)	Mean (SD)
GA ^1^, mean (SD), week	38.27 (1.59)	38.21 (1.71)	38.29 (1.55)	38.31 (1.66)
Birth weight, mean (SD), g	3048.79 (444.34)	3037.62 (458.41)	3052.43 (439.59)	3068.99 (451.9)
Age, mean (SD), years	5.49 (3.63)	5.64 (3.34)	5.44 (3.72)	6.75 (3.71)
	%	%	%	%
Child Sex				
Male	51.49	51.71	51.41	52.00
Female	48.51	48.29	48.59	48.00
Birth order				
1	61.74	63.02	61.32	65.29
2	32.17	31.66	32.34	30.42
≥3	6.09	5.32	6.34	4.29
Maternal age				
<20	2.66	1.59	3.01	1.96
20–24	13.35	12.06	13.76	11.81
25–29	31.77	33.25	31.28	31.68
30–34	35.69	37.60	35.07	37.46
35–39	14.29	13.50	14.55	14.90
≥40	2.25	2.01	2.33	2.20
Smoking during pregnancy	0.08	0.08	0.08	0.07
Alcohol use during pregnancy	0.01	0.01	0.01	0.01
Low income	6.79	5.23	7.30	4.96
Tic disorder	1.02	1.19	0.97	1.32

^1^ gestational age.

**Table 2:** Hazard ratios for TS and tics in offspring of mothers diagnosed with anemia during pregnancy, with consideration of the timing of diagnosis.

Risk of Tic Disorders	Model 1 ^2^		Model 2 ^3^		Model 3 ^4^	
	aHR (95% CI)	P	aHR (95% CI)	P	aHR (95%CI)	P
All exposed vs. nonexposed	1.04 (0.99–1.11)	0.089	1.05 (0.97–1.13)	0.228	1.02 (0.85–1.19)	0.831
Exposed, GA ^1^ ≤12 week vs. nonexposed	1.23 (1.12–1.34)	<0.001	1.16 (1.04–1.28)	0.008	1.06 (0.80–1.32)	0.685
Exposed, GA ^1^ >12 week vs. nonexposed	1.01 (0.93–1.09)	0.813	1.03 (0.99–1.08)	0.103	0.96 (0.80–1.12)	0.661

^1^ gestational age. ^2^ Model 1: clustered on maternal identifier, adjusted for only birth year and sex. ^3^ Model 2: clustered on maternal identifier, adjusted for birth year, sex, low income, maternal age, birth order, gestational age, birth weight, and alcohol use and smoking during pregnancy. ^4^ Model 3: conditional proportional hazard model, adjusted for only birth year and sex, maternal age, birth order, gestational age, birth weight, and alcohol use and smoking during pregnancy. Exactly 64,741 discordant pairs were identified with a total of 145,096 participants in the analysis of Model 3.

## Data Availability

None of the datasets analyzed during this study are publicly available under the regulations of the Department of Health in Taiwan.

## Supplementary Materials

The following are available online at https://www.mdpi.com/article/10.3390/jpm11101038/s1, Table S1: Selected Characteristics by Maternal Anemia Exposure in the Youth Cohort with Exact Number for Group.
