# Supplementary material for: Association of Prenatal Maternal Anemia with Tics and Tourette’s Syndrome in Offspring
Source: J Pers Med. 2021 Oct 17;11(10):1038. doi: 10.3390/jpm11101038 (PMC8541066; doi:10.3390/jpm11101038)
Supplement: Supplementary file 1 [file jpm-11-01038-s001.zip › jpm-1416768-supplementary.pdf]

Supplementary Table S1. Selected Characteristics by Maternal Anemia Exposure in the Youth Cohort with Exact Number for Groups

	**All exposed (n=153,854)**	**Exposed, GA[1] ≤12 week (n=37,832)**	**Exposed, GA[1] >12 week (n =116,022)**	**Unexposed (n=2,014,619)**
	Mean (SD)	Mean (SD)	Mean (SD)	Mean (SD)
GA[1], mean (SD), week	38.27 (1.59)	38.21 (1.71)	38.29 (1.55)	38.31 (1.66)
Birth weight, mean (SD), g	3048.79 (444.34)	3037.62 (458.41)	3052.43 (439.59)	3068.99 (451.9)
Age, mean (SD), years	5.49 (3.63)	5.64 (3.34)	5.44 (3.72)	6.75 (3.71)
Child Sex	N	N	N	N
Male	79213	19563	59650	1047648
Female	74641	18269	56372	966971
Birth order				
1	94991	23842	71149	1315410
2	49500	11977	37523	612784
≥3	9363	2013	7350	86425
Maternal age				
<20	4090	601	3489	39430
20-24	20533	4563	15970	237884
25-29	48873	12578	36295	638248
30-34	54909	14223	40686	754647
35-39	21983	5106	16877	300131
≥40	3466	761	2705	44279
Smoking during pregnancy	125	31	93	1430
Alcohol use during pregnancy	18	5	13	181
Low income	10446	1979	8467	99864
Tic disorder	1575	450	1125	26626
